# Comprehensive molecular and cellular studies suggest avian scutate scales are secondarily derived from feathers, and more distant from reptilian scales

**DOI:** 10.1038/s41598-018-35176-y

**Published:** 2018-11-13

**Authors:** Ping Wu, Yung-Chih Lai, Randall Widelitz, Cheng-Ming Chuong

**Affiliations:** 1 Department of Pathology, Keck School of Medicine, University Southern California, Los Angeles, CA 90033 USA; 2 Integrative Stem Cell Center, China Medical University Hospital, China Medical University, Taichung, 40402 Taiwan; 3 International laboratory for Wound Repair and Regenerative Research, Graduate Institute of Clinical Medicine, National Cheng Kung University, Tainan, Taiwan

## Abstract

Amniote skin appendages such as feathers, hairs and scales, provide thermoregulation, physical protection and display different color patterns to attract a mate or frighten an adversary. A long-standing question is whether “reptile scale” and “avian leg scales” are of the same origin. Understanding the relation between avian feathers, avian scales and reptilian scales will enhance our understanding of skin appendage evolution. We compared the molecular and cellular profiles in chicken feather, chicken scales and alligator scales and found that chicken scutate scales are similar to chicken feathers in morphogenesis at the early placode stage. When we compared the expression of the recently identified feather-specific genes and scale-specific genes in these skin appendages, we found that at the molecular level alligator scales are significantly different from both chicken feathers and chicken scales. Furthermore, we identified a similarly diffuse putative stem cell niche in morphologically similar chicken and alligator scales. These putative stem cells participate in alligator scale regeneration. In contrast, avian feathers have a more condensed stem cell niche, which may be responsible for cycling. Thus, our results suggest that chicken and alligator scales formed independently through convergent evolution.

## Introduction

**Figure 1:**
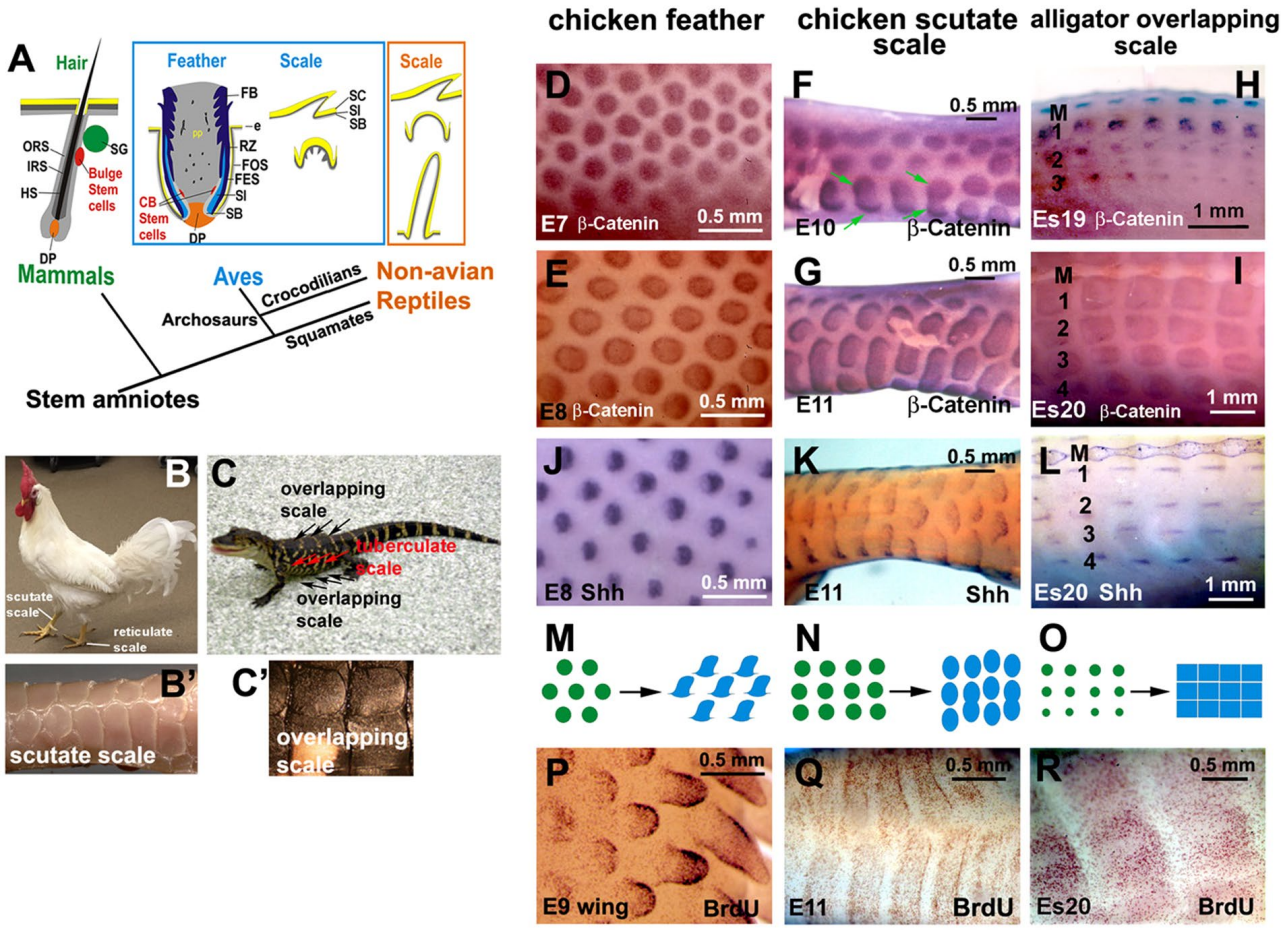
Development of avian and reptilian scales. (**A**) Schematic drawing of the stem cell niche in mammalian hairs and avian feathers. (**B**) Adult chicken showing feathers and scales. (**B’**) Scutate scales. (**C**) Juvenile alligator showing different types of scales. (**C’**) Overlapping scale. D-I, β-catenin whole mount *in situ* hybridization. (**D**) E7 chicken dorsal feather tract (placode stage). (**E**) E8 chicken dorsal feather tract (short bud stage). (**F**) E10 chicken scutate scale (placode stage). Green arrows indicate the fusion of scutate scale placodes. (**G**) E11 chicken scutate scale (short bud stage). (**H**) Es19 alligator overlapping scale (placode stage). (**I**) Es20 alligator overlapping scale (short bud stage). (**J–L**) Shh whole mount *in situ* hybridization. J, E8 chicken dorsal feather tract. (**K**) E11 chicken scutate scale. (**L**) Es20 alligator overlapping scale. (**M–O**) Schematic drawing of skin appendage development. (**M**) Chicken feather, (**N**) chicken scutate scale, (**O**) alligator overlapping scale. (**P**–**R**) Whole mount BrdU staining. (**P**) Feather buds in an E9 chicken wing showed different feather developmental stages, from short buds to long buds. (**Q**) E11 chicken scutate scale. (**R**) Es20 alligator overlapping scale. Note the feathers have a broader localized growth zone than scales. CB, collar bulge; DP, dermal papilla; e, epidermis; FB; feather barb ridge; FES, feather sheath; FOS, feather follicle sheath; HS, hair shaft; IRS, inner root sheath; M, dorsal middle line of alligator embryo; ORS, outer root sheath; RZ, ramogenic zone; SG, sebaceous gland; SB, stratum basal; SC, stratum corneum; SI, stratum intermedium; 1, 2, 3, 4 indicate the row number with 1 closest to the middle of the dorsal region.

Amniotes exhibit different types of skin appendages including scales, feathers, hairs, teeth, beaks and claws. Reptile scales represent the basal type of amniote skin appendages from which feathers and hairs were thought to have evolved (Fig. 1A)^1–3^. Reptile scales, as found on alligators, have a flattened, overlapping appearance on dorsal regions, as well as on the belly and leg of the animal (Fig. 1C,C’). Dome shaped tuberculate scales are formed on the lateral side of the body (Fig. 1C)^4^. Birds not only have feathers on their body but also have scales on their feet, which includes two main types: the overlapping scutate scales, which form in the metatarsal region, and the dome shaped reticulate scales positioned on the underside of the foot (Fig. 1B,B’)^5^. Morphologically, avian scutate scales are similar to crocodilian scales with overlapping skin folds, whereas avian reticulate scales are similar to reptilian tuberculate scales. Here we explore the relationship between chicken scutate scales and alligator overlapping scales.

The relationship among avian feathers, avian scales and reptilian scales has fascinated scientists for decades. Understanding this relationship may help to unveil the origin of avian feathers, which eventually enabled birds to fly and venture into their new eco-system. Currently there are two hypotheses explaining the origin of avian feathers. The first hypothesis suggests that all ectodermal organs, including feathers, scales, teeth, etc, evolved independently from a common primitive placode^6^. The second concept is that avian feathers evolved from primitive reptilian scales^7^. The evolutionary origin of avian scales is also controversial. For its origin, there are two different views. The first view is that avian scales are the remnant of reptilian scales^8,9^. The second view is that avian scales are the secondary derivatives from avian feathers^10,11^. Some paleontological studies support this view^12,13^. Feathered feet are also seen in some extant birds, such as golden eagles and domestic pigeons. Here we take a molecular and cellular approach to study this issue.

Molecular and cellular studies have been used to understand the evolution of amniote skin appendages in extant reptiles and birds. Shh and Bmp2 signaling has been found to form a functionally conserved developmental signaling module in archosaur epidermal appendage development^14^. Using histological and molecular techniques, reptilian scales (crocodiles and snakes) were shown to exhibit a placode configuration, which suggests that hairs, feathers and scales of extant amniotes are modified from ancient reptilian scale placodes^15^. Comparative genomic studies identified conserved non-exonic elements that suggest exceptional regulatory innovation in the archosaur lineage. Also, the presence of feather development genes predates the appearance of feathers, signifying that avian dinosaur ancestors already had the non-keratin molecular toolkit necessary to make feathers^16^. Recently, through genomic and functional studies in embryonic chicken skin, we identified mesenchymal feather-associated genes and proposed that multiple regulatory modules were involved in scale to feather evolution. In addition, we found that candidate molecules also caused the formation of localized growth zones (LoGZ) and invaginations in alligator skin^17^.

Avian feathers and mammalian hairs share many common characteristics, including the topology of their stem cells and transient amplifying cells (TA cells), the presence of a dermal papilla, as well as proximal - distal polarity^18^. Feathers and hairs are periodically replaced throughout the life of each animal^9,19^. The cycling relies on activation of stem cells in a supportive niche within their follicular structure^20,21^. In hair follicles, the stem cell compartment is localized within the bulge^22^, whereas in the feather follicle, stem cells are located within the collar bulge^23^ (Fig. 1A). In contrast both avian and reptilian scales are not follicular structures and some reptiles, such as snakes, will shed their skin all at once, while lizards shed their skin in patches as they grow. Molting in Alligators and Crocodiles can occur in individual scales.

Bromodeoxyuridine (BrdU or 5-BrdU) retention was successfully used to locate slow cycling cells in hair^24^ and feather follicles^21^. Since stem cells do not often enter the proliferative phase of the cell cycle, once labeled with experimentally detectable thymidine analogs, they are expected to retain the label for long periods of time and are named label retaining cells (LRCs). Further studies using EGFP-tagged histone 2B (H2BEGFP) and the K15 promoter were used to identify stem cell populations in hair follicles^25–27^ and proved that LRCs in the hair follicle are multipotential stem cells. However, the location of LRCs (or the putative stem cells) in both avian and reptilian scales remains unknown.

In this paper, we examine similarities and differences in cell behavior and molecular profiles among chicken feathers, chicken scales and alligator scales during development and regeneration. We find putative stem cell configurations diffusely clustered in the hinge regions of alligator overlapping scales and chicken scutate scales. We then demonstrate whether these specified putative stem cells participate in scale regeneration. However, at the transcriptome level, we demonstrate alligator scales are significantly different from both chicken feathers and chicken scales, suggesting that they evolved independently.

## Results

### The development of chicken scutate scales and alligator overlapping scales use diffuse cell proliferation and signaling molecule expression

We wondered what mechanism differentially regulated the morphogenesis of avian feathers versus avian scales and reptilian scales. We hypothesized that signaling molecule expression and cell proliferation might display different distributions in feathers and different shaped scales. To elucidate this, we investigated molecular expression and cell proliferation during their early skin appendage development stages (Fig. 1D–R). Chicken skin appendage formation appears heterogeneous with feathers developing earlier than scutate scales. E7 (embryonic day 7, stage 31) feather forming skin is equivalent to E9 (stage 35) scale forming skin as both are in the placode stage. We used whole mount *in situ* hybridization (WMIH) of β-catenin to show the placode (Fig. 1D,F,H), and short bud stage (Fig. 1E,G,I) of each of these skin appendages. We then performed *in situ* hybridization of Shh (Fig. 1J,K,L) to show the short bud stages. Chicken feathers developed from placodes and form short buds (Fig. 1D,E,J). Alligator overlapping scales first formed circular spots which then expanded to become square in shape (Fig. 1H,I). WMIH of Shh appeared as a rostral-caudal stripe in the center of each scale primordia (Fig. 1L). However, chicken scutate scales started from spots but then some spots merged to form the horizontally enlarged scale buds (arrows in Fig. 1F,G). Thus, we found that the development of chicken scutate scales is different from that of both chicken feathers and alligator scales, but the shape of chicken scutate scale placodes is more similar to chicken feathers (Fig. 1M–O).

When we compared cell proliferation among feathers and different scale types, we found that only the feather primordia showed a clear, localized growth zone (Fig. 1P). The cell proliferation of chicken scutate scales and alligator scales were more diffuse (Fig. 1Q,R). The diffuse, asymmetric pattern of cell proliferation along the anterior-posterior axis was observed in both chicken scutate scales and alligator scales (Fig. 1Q,R).

These results suggest that some of avian scutate scale development relies upon primordia fusion. The development of scutate scales started from a round shaped placode (similar to a feather placode) and then underwent a horizontal expansion process. Avian scales and reptilian scales display diffuse patterns of molecular expression and cell proliferation, when compared to that of avian feathers.

### RNA-seq analysis reveals that embryonic chicken scutate scales and alligator overlapping scales have different molecular profiles than embryonic chicken feathers

Previous studies from our lab have identified 102 *feather-associated genes* in developing feather dermis and *170 scale-associated genes* through the examination of transcriptome data of developing chicken feathers and scales including both region-independent genes and development-dependent genes^17^. We examined whether alligator skin expresses the *feather-associated genes* or *scale-associated genes* to determine at the molecular level if alligator scales are closer to avian feathers or avian scales in development. We conducted RNA-seq using the dermis of Es19 (placode stage) and Es22 (bud stage) alligator dorsal and ventral overlapping scales. We compared these RNA-seq data with those of chicken feather- or scale-associated genes.

**Figure 2:**
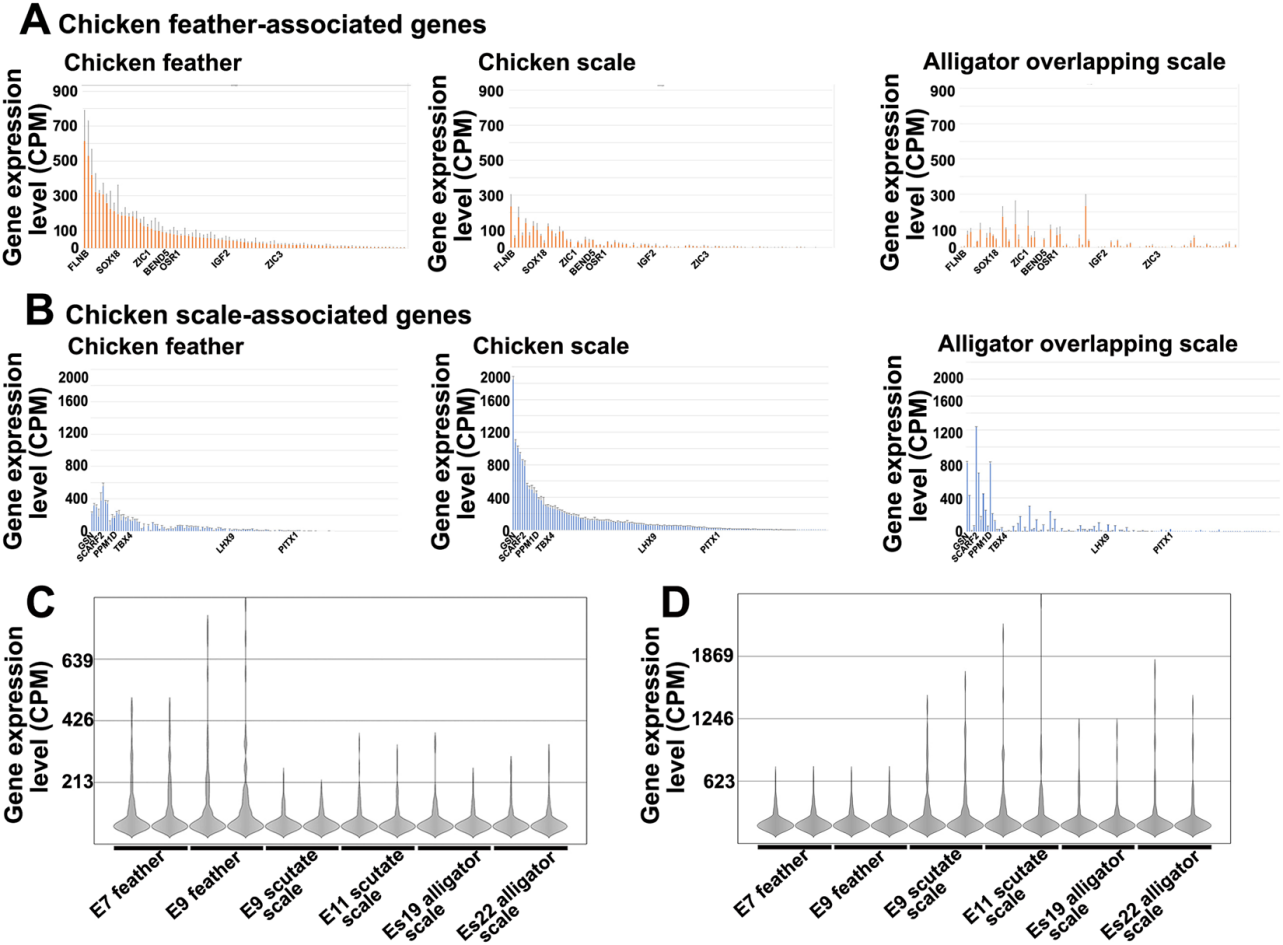
RNA-seq analysis of embryonic chicken feathers, chicken scutate scales and alligator overlapping scales. (**A**) Comparing the chicken *feather-associated gene* expression profile among chicken feathers (E7 and E9), chicken scutate scales (E9 and E11) and alligator overlapping scales (Es19 and Es22). The X-axis is the average CPM (count per million) value of *feather-associated genes* (Y-axis) from high to low in feather samples (left panel). Chicken scale (middle panel) and alligator scale (right panel) expression levels of *feather-associated gene* are shown. (**B**) Comparing the chicken *scale-associated gene* expression profile among chicken feathers (E7 and E9), chicken scutate scales (E9 and E11) and alligator overlapping scales (Es19 and Es22). The X-axis is the average CPM (count per million) value of *scale-associated genes* (Y-axis) from high to low in chicken scutate scale samples (middle panel). Chicken feather (left panel) and alligator scale (right panel) expression levels of *scale-associated gene* are shown. (**C**) Violin plots showing the chicken *feather-associated genes* are down-regulated in chicken scales and alligator scales. (**D**) Violin plot analysis showing the chicken *scale-associated genes* are down-regulated in both chicken feathers and alligator scales.

Among the 102 *feather-associated genes*^17^, 86 have been annotated in the alligator genome^28^ (Fig. S1). After aligning the reads to the alligator genome, the expression level of these 89 genes was calculated. We first arranged their average CPM (count per million) values in chicken feather samples from high to low (Fig. 2A, left panel) and compared the CPM value in chicken scale samples (Fig. 2A, middle panel) and alligator scale samples (Fig. 2A, right panel). The expression levels of these 86 genes in chicken scutate scales is much lower than that found in feathers, as expected (Fig. 2A, middle panel). When we examined their expression levels in alligator scale samples. We found that that the expression of chicken feather-associated genes in alligator scales was distinct from that seen in both chicken feathers and chicken scales (Fig. 2A, right panel).

We then examined the *scale-associated genes* using the same strategy (Fig. 2B). Among the 170 chicken *scale-associated genes*^17^, 135 have been annotated in the alligator genome^28^ (Fig. S2), so we focused our analysis on these 135 genes. As expected, these chicken scale-associated genes were expressed to much higher levels in chicken scales (Fig. 2B, middle panel) than in chicken feathers (Fig. 2B, left panel). When we examined the expression levels of these genes in alligator scale samples (Fig. 2B, right panel), most of the genes did not show similar expression levels seen in chicken scale samples (Fig. 2B, middle panel). These results imply that even though chicken and alligator scales share some morphological similarities, their molecular expression profiles are distinctly different.

We performed a Violin plot analysis to visualize the distribution of the normalized expression levels. It helps to highlight the most highly expressed *feather-associated genes* in chicken feathers. We found that *feather-associated gene* expression in chicken scales and alligator scales are down-regulated compared to chicken feathers (Fig. 2C). The Violin plot analysis of chicken *scale-associated genes* showed that they were down-regulated in both chicken feather and alligator scale samples (Fig. 2D). This analysis suggests that avian feathers require novel molecular circuits distinct from those used to produce avian and reptilian scales.

### Adult chicken and alligator scales have diffuse LRCs

**Figure 3:**
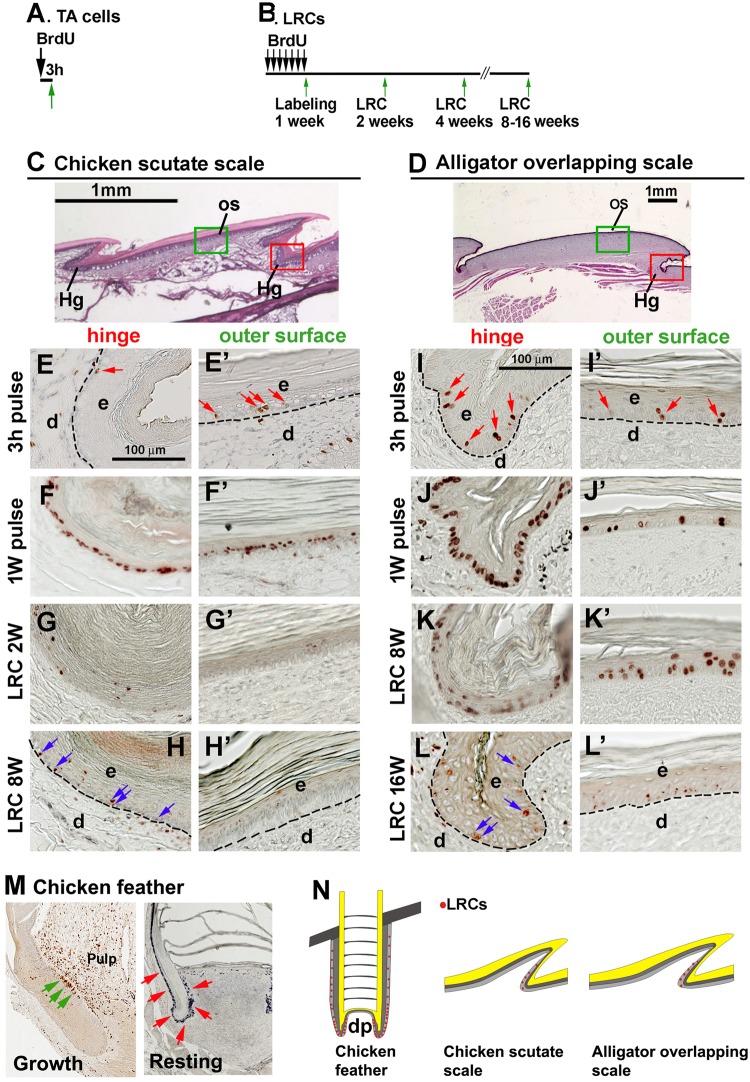
The topology of putative stem cells in chicken scutate scales and alligator overlapping scales. (**A**) Strategy of TA cell labeling. (**B**) Strategy of LRC labeling. (**C**) H&E staining of a chicken scutate scale. (**D**) H&E staining of an alligator overlapping scale. The red and green rectangular boxes in panel C and D indicate the hinge region and outer surface region in chicken and alligator scales. (**E–H’**) TA and LRCs in chicken scutate scales. (**E**) BrdU 3-hour pulse labeling. Red arrows indicate BrdU positive cells. (**F**) BrdU 1-week labeling. (**G**) A 2-week chase period after 1-week of labeling. (**H**) An 8-week chase period after 1-week of labeling. Blue arrows indicate the LRCs in the hinge region. (**I–L’**) TA and LRCs in alligator overlapping scales. I. 3-hour BrdU pulse labeling. Red arrows indicate BrdU positive cells. (**J**) BrdU labeling for 1-week. (**K**) An 8-week chase period after 1-week of labeling. (**L**) A 16-week chase period after 1-week of labeling. Blue arrows indicate the LRCs in the hinge region. Note that LRCs exist in the hinge regions but are negative in the outer surface in both chicken scutate and alligator overlapping scales. (**M**) LRCs in chicken feathers. Left panel, growth phase, green arrows indicate the LRCs in the feather bulge region. Right panel, resting phase, red arrows indicate the LRCs in epidermis surrounding the dermal papilla (papilla ectoderm) and in the follicle sheath. (**N**) Schematic drawing showing the distribution of LRCs (red dots). The dotted lines separate epidermis and dermis. d, dermis; e, epidermis; Hg, hinge; OS, outer surface.

We asked whether stem cells are localized within the stem cell niche in scales. If this is the case, are the properties of their stem cell niche different from that found in feathers? To answer this question, we used a 3-hour BrdU pulse labeling to identify the transit amplifying cells (TA cells) (Fig. 3A) and the BrdU label retention (LRC) method to locate the slow cycling cells which are putative stem cells (Fig. 3B).

H&E staining showed that the morphology of chicken scutate scales and alligator overlapping scales are similar. Both have an outer surface and an inner surface connected by the hinge region (Fig. 3C,D). We focused on the hinge and outer surface area.

We first sought to identify the configuration of stem cells and TA cells in adult chicken scutate scales by comparing their outer surface and hinge regions (Fig. 3E–H’). After a 3 h pulse labeling, BrdU positive cells were randomly distributed in the epidermis of both the hinge and outer surface regions (Fig. 3E,E’, red arrows). After a 1-week BrdU labeling period, most keratinocytes in the basal layer (95% in the hinge region and 80% in the surface epithelium) were BrdU positive (Fig. 3F,F’). This was followed by a 2-week chase period in which dividing cells lost their label. LRCs were seldom detected in the basal layer of the epidermis but were found distributed in the hinge region of scutate scales (Fig. 3G). However, no LRCs were detected in the basal layer of the outer surface epidermis (Fig. 3G’). Similar numbers of LRCs in the hinge (blue arrows) were found even after an 8-week chase period (Fig. 3H,H’).

In alligator overlapping scales, a short term 3-hour BrdU labeling identified proliferating cells randomly distributed in the epidermis of both the hinge and outer surface (Fig. 3I,I’, red arrows). After BrdU labeling for 1-week, 95% of basal layer cells in the hinge region (Fig. 3J) but only 15% of cells in the outer surface region (Fig. 3J’) are BrdU positive. These data suggest that the hinge epidermis has more cell proliferation than the surface region. The 3-hour pulse labeling was insufficient to reflect cell proliferation differences between outer surface and hinge regions. After an 8-week chase period there are LRCs in both the hinge and outer surface (Fig. 3K,K’). However, after a 16-week chase, the LRCs are only detected within the hinge region (Fig. 3L, blue arrows). Only speckled BrdU labeling is detected in the surface region (Fig. 3L’). Those cells with a speckled appearance are considered to have more divisions which dilute the BrdU labeling and therefore are not counted as LRCs^29^.

Compared to chicken feathers, whose LRCs are clustered and localized in the collar bulge area during the growth phase (Fig. 3M, left panel, green arrows) and in the papilla ectoderm and the lower follicle sheath during resting phase (Fig. 3M, right panel, red arrows), LRCs in both chicken scutate scales and alligator overlapping scales are diffusely distributed in the hinge region.

These results suggest that the stem cell niche configuration of both avian and reptilian scales is different from that found in avian feathers. LRCs in both chicken and alligator scales are diffusely distributed in the hinge region. It is reasonable to find the stem cell niche in the protected hinge region. Whereas feathers have a stem cell ring in the collar bulge during growth phase. Even in the resting phase, clustered LRCs can be detected in the papilla ectoderm and the lower follicle sheath (Fig. 3M). We summarize the location of LRCs in chicken feathers, chicken scutate scales and alligator overlapping scales in Fig. 3N.

### The stem cell marker, K15, is expressed in the hinge of both chicken scutate and alligator overlapping scales

The intermediate filament keratin 15 (K15) has been used as a hair follicle stem cell marker^30,31^. To detect the expression pattern of K15 in different amniote skin appendages, we generated chicken-specific and alligator-specific K15 antisense probes. Another alpha keratin, K75, which is not associated with stem cells, was used for comparison.

**Figure 4:**
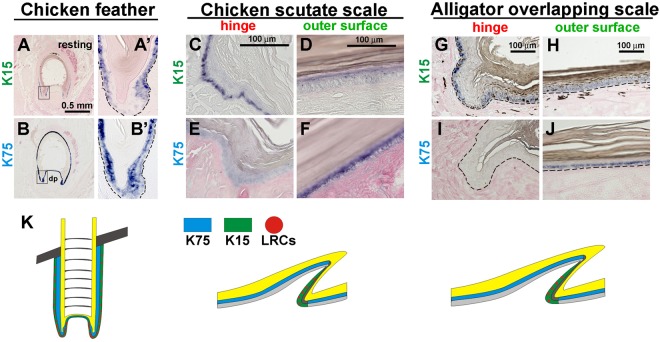
Expression of the stem cell marker, K15 and differentiation marker, K75 in chicken and alligator scales. (**A,A’**) Expression of K15 in resting phase feather follicles. (**B,B’**) Expression of K75 in resting phase feather follicles. (**C–F**) Chicken scutate scales. (**C,D**) Expression of K15 in chicken scutate scales. (**C**) Hinge; (**D**) outer surface. (**E,F**) Expression of K75 in chicken scutate scales. (**E**) Hinge; (**F**) outer surface. (**G**–**J**) Alligator overlapping scales. (**G,H**) Expression of K15 in alligator overlapping scales. (**G**) Hinge; (**H**) outer surface. (**I,J**) Expression of K75 in alligator overlapping scales. (**I**) Hinge; (**J**) outer surface. Note K15 is positive in the hinge basal layer (**C**,**G**) whereas K75 is positive in the outer surface suprabasal layer (**F,J**). (**K**) Schematic drawing of the distribution of K15 (green), K75 (blue) and LRCs (red dot) in feathers (resting stage) and scales. dp, dermal papilla.

We first examined the expression of K15 and K75 in chicken adult feathers. At resting stage, both K15 and K75 are expressed in feather epidermis. K15 is expressed in the basal layer (Fig. 4A,A’), whereas K75 is expressed in the supra-basal layer (Fig. 4B,B’). LRCs in resting stage feathers were localized in the basal layer of the epidermis (Fig. 3N, left panel), which is K15 positive (Fig. 4A’). In comparison, K15 is strongly expressed in the hinge basal layer (Fig. 4C) but faintly expressed in the outer surface in adult scutate scales, (Fig. 4D), whereas K75 is expressed in the supra-basal layer in the surface (Fig. 4F), but not in the hinge region (Fig. 4E).

We further investigated the expression of K15 in alligator overlapping scales. K15 is expressed in the basal layers of the hinge (Fig. 4G) but faintly in the surface region (Fig. 4H). K75 is expressed in the supra-basal layers of the surface region (Fig. 4J) but not in the hinge (Fig. 4I).

We conclude that the hinge of both chicken scutate scales and alligator overlapping scales are K15 positive. Whereas K75 is expressed in the supra-basal layer of the surface which is complementary to K15 (Fig. 4K). The hinge region of scales demonstrates a unique architecture facet which is used to preserve putative stem cells. Whether these putative stem cells participate in the repair and regeneration process is interesting to pursue as we reported in the following study.

### Wound healing/regeneration abilities in chicken and alligator scales

To test the regenerative abilities of avian and reptilian scales, a piece of skin (1 cm × 1 cm) (including both epidermis and dermis) covering several scales was surgically removed from the adult chicken leg and juvenile alligator body.

**Figure 5:**
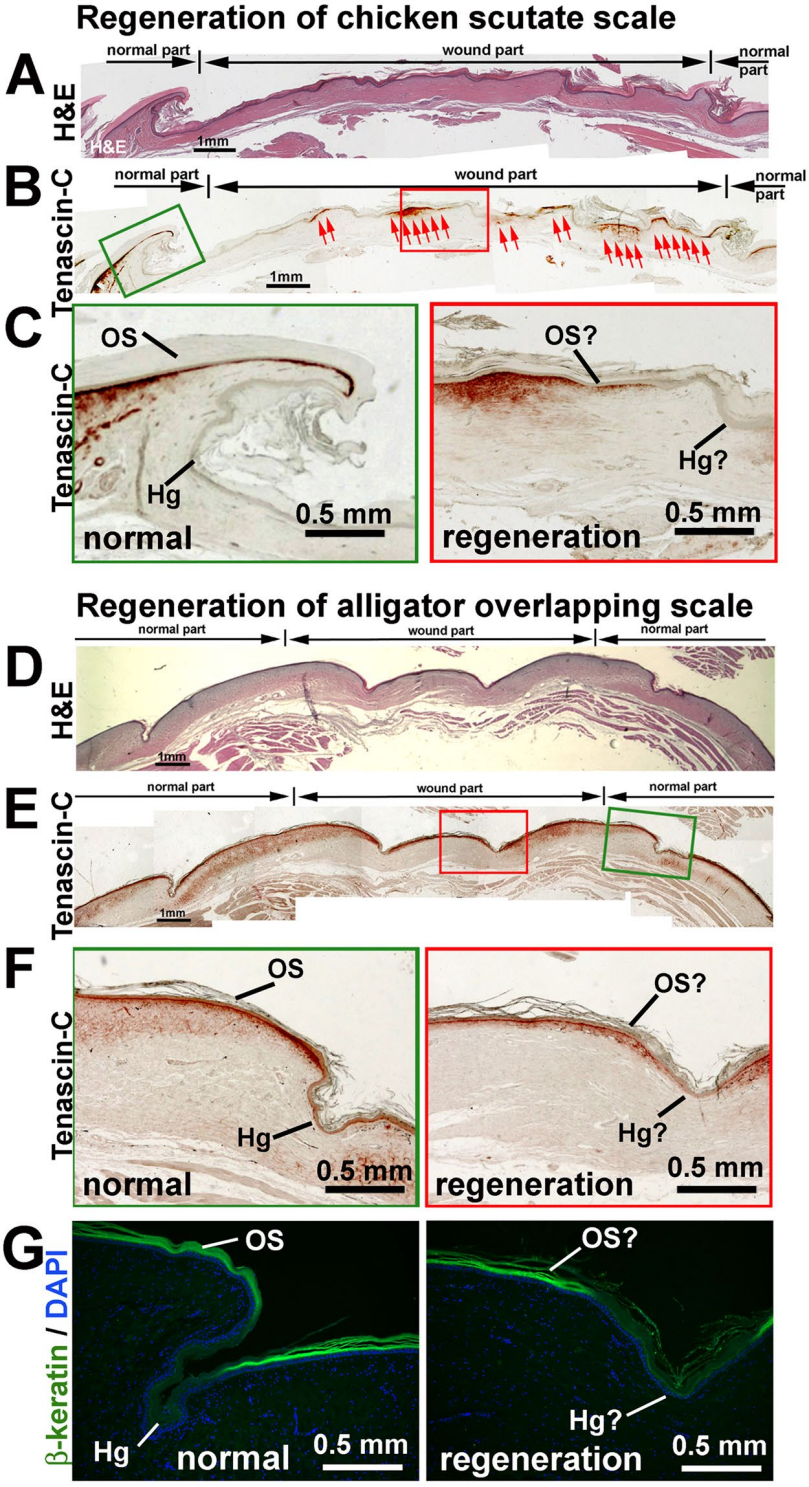
Regenerative power of avian and reptilian scales. (**A–C**) Wounded chicken scutate scales after 4 months of healing. (**A**) H&E staining. (**B**) Tenascin-C staining. (**C**) Higher magnification of (**B**). Left, normal scale beside the wound site. Right. regenerated scale. (**D–F**) Wounded alligator scale after 4 months of healing. (**D**) H&E staining. (**E**) Tenascin-C staining. (**F**) higher magnification of (**E**) (**G**) β-keratin staining. (**F**) and (**G**) Left, normal scale beside the wound site; Right, regenerated scale. Hg, hinge; OS, outer surface.

When the wound was made in a chicken scutate scale forming region, after 16 weeks, the wound site was covered with scales. However, overlapping scales formed incompletely (Fig. 5A, H&E staining, the wound region compared to the normal unwounded part). Regenerated scales showed a periodic Tenascin-C expression pattern (red arrows in Fig. 5B). In normal scale, Tenascin-C is expressed in the dermis of the surface region (Fig. 5C, left panel). Morphologically these regenerated scales showed a slightly overlapping pattern. It also showed positive expression of Tenascin-C in the outer surface but is negative in the “hinge” (Fig. 5C, right panel). This result suggests that avian scutate scales have a partial regenerative ability, as it could form the “outer surface” and “hinge” but could not fully form overlapping scale structures.

We then checked the regeneration power of alligator overlapping scales. Similar to chicken scales, the wound area was covered by scales after 16 weeks of regeneration (Fig. 5D). H&E staining of sections showed that the regenerated skin covering the wound did not form overlapping scale structures because the morphology of the surface and hinge regions are altered (Fig. 5D). As in chicken scutate scales, Tenascin-C is expressed in the dermis of the surface region but is negative in the hinge region of alligator overlapping scales (Fig. 5F, left panel). The regenerated scales display a similar Tenascin-C expression pattern although it is thinner, in the “surface” area and absent from the “hinge” region (Fig. 5E, enlarged in Fig. 5F, right panel). The regenerated “hinge” region shows that β-keratin (also known as corneous beta proteins^32^) expression is undetectable (Fig. 5G, right panel). In normal alligator overlapping scale, β-keratin expressed is only located in the outer surface, but not in the hinge region (Fig. 5G, left panel)^8^. This result suggests that alligator overlapping scales have partial or limited regeneration power.

We conclude that unlike the robust regenerative power of feathers and hairs, both avian and reptilian scales have repair but limited regenerative abilities. However, avian scutate scales and reptilian overlapping scales can regenerate “surface” and “hinge” like architecture.

### LRCs in the hinge participate in wound healing of alligator overlapping scales

**Figure 6:**
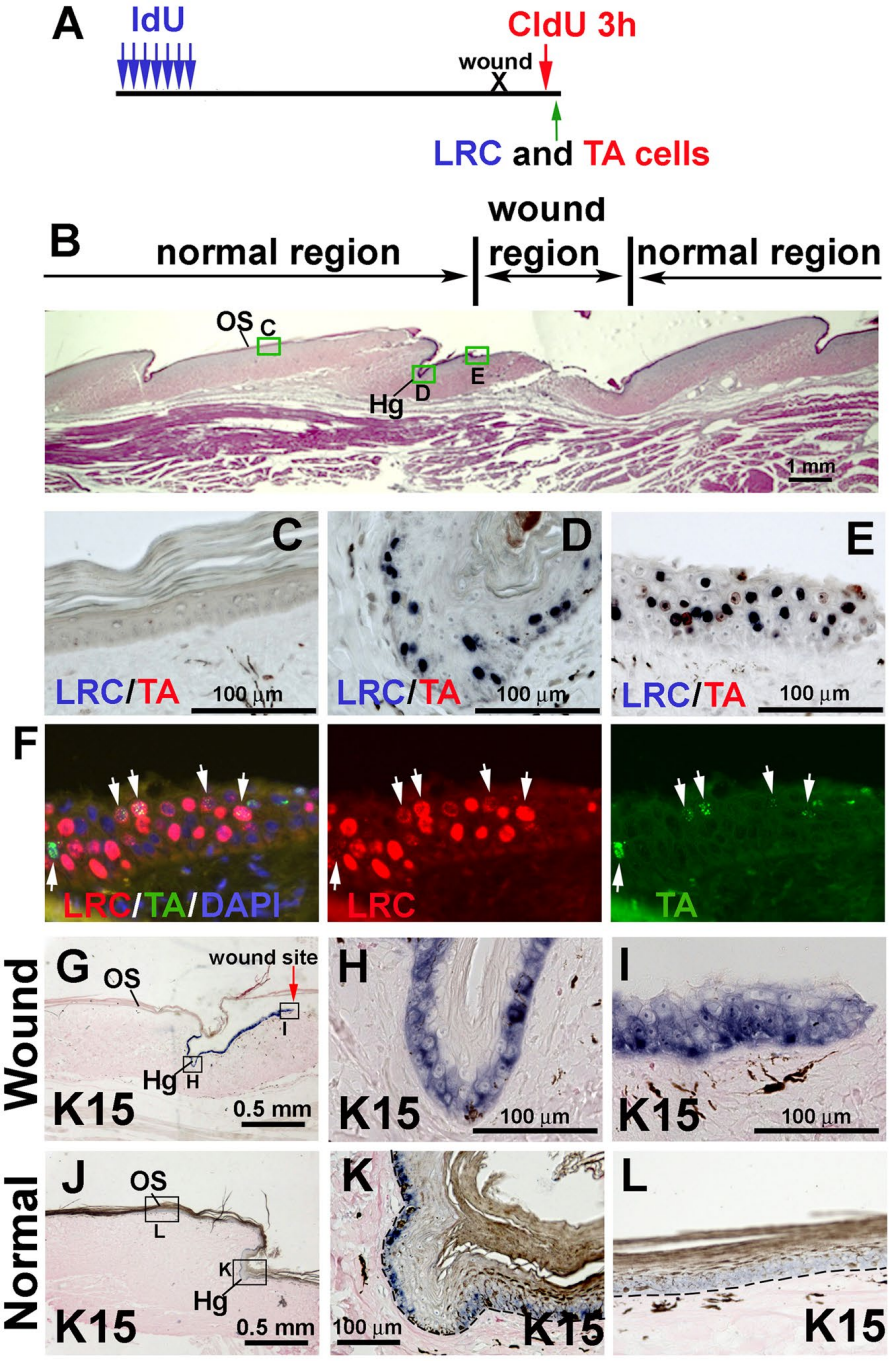
Cell behavior after wounding of reptilian overlapping scales. (**A**) Strategy of labeling LRCs with IdU and TA cells with CldU to study cell dynamics after wounding. (**B**) H&E staining after 4 days of wound healing. The rectangular blocks show the region for CldU and IdU staining enlarged in panel (**C–E**). (**C**) Outer surface of a scale beside the wound site. (**D**) Hinge of a scale closest to the wound site. (**E**) Outer surface region in the wound site. Note the accumulation of LRCs (blue color) in (**D**) and (**E**). (**F**) Fluorescent immunostaining of IdU (LRCs, red color) and CldU (TA cells, green color). Left, Triple staining of LRCs, TA cells and DAPI (blue color); Middle, LRCs only; Right, TA cells only. White arrows indicate IdU and CldU double positive cells which suggests the LRCs are in a proliferating status. (**G**) K15 *in situ* hybridization showing increased K15 expression in epidermis near the wound site. (**H**) Hinge closest to the wound site. I, outer surface in the wound site. (**J**) K15 *in situ* hybridization showing the normal K15 expression in an overlapping scale. (**K**) Hinge. (**L**) Outer surface. Hg, hinge. OS, outer surface.

To investigate whether the LRCs take part in the wound healing process, we used a 5-Iodo-2-deoxyuridine (IdU) / 5-Chloro-2-deoxyuridine (CldU) double labeling method. We have used this method to study the LRCs and TA cells in alligator dental lamina^33^. To do this, juvenile alligators were labeled with IdU for 1 week, then chased for 16 weeks. A wound was made in the overlapping scales and wound healing was assessed 4 days later. A 3-hour CldU pulse was administered before the animal was euthanized. Thus, we can monitor both LRCs and TA cells in the same sample (Fig. 6A).

H&E staining showed that the wound was not re-epithelialized by 4 days after wounding (Fig. 6B). IdU (blue color) and CldU (red color) double staining of three areas are shown in Fig. 6C–E. The outer surface region in a normal scale close to the wound are both IdU/CldU negative (Fig. 6C). The hinge close to the wound has multiple IdU positive cells but no CldU positive cells (Fig. 6D). To our surprise, the outer surface adjacent to the wound is full of LRCs and some TA cells (Fig. 6E). Double fluorescent staining showed that some of the LRCs (red color) have undergone at least one round of proliferation (TA cells, green color) (Fig. 6F, white arrows). This result demonstrates that LRCs accumulate in the wound area in both the outer surface and hinge. Some of the LRCs start to proliferate within the wound site.

We further examined whether K15 is expressed within the wound site. K15 expression is restricted to the basal layer cells of the hinge region but not in the outer surface under normal conditions (Fig. 6J–L). However, in the wounded scale, K15 expression extends to the outer surface close to the wound (Fig. 6G–I). The coincident accumulation of LRCs and extension of K15 expression in the wound site suggests that the putative stem cells participate in wound healing and may play an important role in the healing process.

## Discussion

The origin of avian scales is controversial. One view is that avian scales are homologues of non-avian reptile scales. However, it also has been hypothesized that bird scutate scales appeared later in evolution and are secondary structures derived from feathers^10,11^. We performed a number of experiments to compare the properties of avian scales and reptilian scales to resolve this issue. On one hand, we showed that both chicken scutate scale and alligator overlapping scale development have diffuse β-Catenin and Shh expression and form a diffuse LoGZ, which is different from the observed focal pattern of gene expression and LoGZ formation in chicken feathers (Fig. 1D–R). On the other hand, we observed that some of chicken scutate scales form through a fusion of small round shaped placodes (Fig. 1F), whereas alligator scale morphogenesis did not go through this fusion process (Fig. 1H). Furthermore, we found avian and reptilian scales exhibit these genes with distinct patterns (Fig. 1F,G,K versus H,I,L), suggesting that they are differentially regulated. Our results indicate that avian scales and reptilian scales may use different molecular circuits to regulate development and morphogenesis, which imply that they have different origins. Our data support the view that avian scales are secondary structures derived from feathers^10,11^.

Another property we evaluated was the configuration of the stem cell compartment in these different skin appendages. The hair and feather follicle provides a niche to protect the stem cells and also to configure the lineage of their progeny. Understanding the homeostasis of stem/TA/differentiated cells in different skin appendages is critical to appreciating differences and similarities in the morphogenesis of different skin appendages. The growth of hair and feather follicles is maintained by stem cells. Hair bulge stem cells and feather collar bulge stem cells were originally discovered using the label retention method^21,24,34^. K15 is observed enriched in the hair follicle bulge region where multipotential epidermal stem cells are located and is used as a marker of hair follicle stem cells^30,31^. Transgenic mice with a K15 promoter driving the expression of a reporter gene demonstrated that the K15 positive epidermal cells have stem cell characteristics^25,26^. Both mammalian hairs and avian feathers have a robust ability to regenerate through normal cycling. Cycling can also be induced upon plucking. Mice display a progressive hair wave during regeneration following hair plucking^35–37^. Mice also demonstrate *de novo* hair regeneration following the formation of large wounds^38^. Hair follicle and IFE stem cells make distinct contributions to regeneration in response to mouse skin wounds^39^.

Due to a lack of powerful genetic tools to study stem cells in bird and reptile skin, we used label retention to identify putative and compare the location of stem cells in avian and reptilian scales. These studies revealed that the LRCs are located in the hinge of avian scutate scales and reptilian overlapping scales (Fig. 3N). Morphologically, the hinge region has a thinner stratum corneum which may be required for reptilian motility. The hinge regions of avian and reptilian scales express different keratins from the surface regions which implies that these regions may have different cell behaviors. We evaluated the temporal and spatial distribution pattern of K15, a stem cell marker, during normal scale development, in mature scales, and in injured mature epidermis. We observed the accumulation of LRCs in wound sites in the alligator overlapping scales, and the accompanying K15 expression domain extension. Our result suggests that the hinge putative stem cells actively contribute to epidermal regeneration during wound healing.

We observed differential retention time of the BrdU label between chicken scutate scales and alligator overlapping scales. In chicken scutate scales, more than 95% of hinge basal layer cells are labeled after 1-week of BrdU treatment (1-week pulse) (Fig. 3F). Only a few of these BrdU positive cells are retained after a 2-week chase (LRC 2-weeks) (Fig. 3G). Similar numbers of LRCs are observed after an 8-week chase period (Fig. 3H). However, in the alligator overlapping scales 40% hinge basal layer cells are BrdU positive after an 8-week chase (Fig. 3K). The difference in the rate of LRC disappearance in the hinge region may reflect a differential metabolic rate between birds and reptiles.

Scales in squamates, in contrast to hairs and feathers, have limited regenerative abilities. We have shown this to be true in the green anole body and tail scales and green iguana^40^. However, the cellular and molecular basis of scale regeneration remains elusive, either at the single scale level or the coordinated response to large skin wounds. Is a specific configuration of stem/TA cells required to form a sustainable regenerative unit containing self-renewing multipotent stem cells? Does the lack of a follicle structure in scales prevent their ability to regenerate? Can this ability be restored by reprogramming during tissue regeneration?

Other labs have also explored properties of scales and found that crocodilian and turtle scales share similarities^41^. This paper highlights that proliferation of these scales differs from those found in leidosaurians which show higher proliferation rates in the outer scale surface. In the chick the length of the G-phase of the proliferation cycle was found to increase in the inner surface region as embryonic development progressed^42^. Also, a lineage of basal cells was found aligned along the avian scale axis at day 10 which then showed polarized divisions at day 11 producing suprabasal cells^43^.

Our result showed that both avian scutate and reptilian overlapping scales have limited regeneration abilities. They each could regenerate surface and hinge-like regions but did not produce regions that overlapped neighboring scales. In response to wound injury, these putative stem cells could move and accumulate within the wound site to take part in the healing and regeneration. However, unlike the hair and feather which have follicular structures with the localized stem cells within their specific niche, these putative scale stem cells are diffusely distributed which may be related to their weak regenerative ability. Another example of robust ectodermal organ regeneration is found in the alligator tooth. In alligator teeth, we also found focally localized LRCs in the “bulge” of the dental lamina^33^, the stem cell niche for new tooth generation. In this animal model, the dental lamina is present in the lingual side of each functional and replacement tooth localized within a single tooth family unit. Our current scale regeneration result indicates that the diffuse putative stem cells in the hinge may be responsible for the observed limited regeneration abilities.

Overall, our results demonstrate in embryonic development, the gene expression profile in alligator scale is very different from both chicken feathers and scales. The shape of the placode for chicken scutate scales is closer to that of the feather than alligator overlapping scales which suggests that avian scutate scales do not have the same origin as reptile overlapping scales. However, in adults, there is a diffuse putative stem cell niche localized within the hinge of both chicken and alligator scales suggesting these structures form through convergent evolution. In contrast, avian feathers have a more condensed stem cell niche which may be responsible for feather morphogenesis and cycling.

## Methods

### Juvenile alligators and adult chickens

We used adult chickens and juvenile alligators (6-month to 1-year-old) for TA and LRC labeling. Alligator eggs were collected from the Rockefeller Wildlife Refuge in Louisiana. Eggs were transported to USC and incubated at 30 °C. Embryo staging was according to^44^. All procedures were approved by the local Institutional Animal Care and Use Committee at the University of Southern California, and the Louisiana Department of Wildlife and Fisheries. The methods were carried out in accordance with the relevant guidelines and regulations.

### RNA-seq analysis of developing alligator overlapping scale

To perform RNA-seq analysis on developing alligator overlapping scales, we collected skin from Es19 and Es22 alligator dorsal and ventral overlapping scales. Epidermis and dermis were separated by treating with 2×CMF solution. RNA from dermis was extracted using Trizol reagent (Invitrogen). 1–2 ug of total RNA from each sample was used to construct an RNA-seq library using TruSeq RNA sample preparation v2 kit (Illumina). Sequencing (50 cycles single read) was performed using Hi-seq2000 at the USC Epigenome Center. Replicate samples from each region and stage were prepared. The full datasets have been submitted to the NCBI Gene Expression Omnibus (GEO).

### Transcriptome comparison of embryonic chicken feathers, scutate scales and alligator overlapping scales

RNA-seq analysis of chicken feathers and scutate scales are from^17^. For alligator skin RNA-seq analysis, the raw reads from embryonic alligator skin were aligned with the alligator genome^28^ using STAR 2.4.1d. Gene expression levels were estimated using CPM (count per million) with TMM normalization.

### Pulse BrdU labeling and identification of label retaining cells

For the 3 hour pulse labelling of juvenile alligators and adult chickens, BrdU was injected intraperitoneally at 50 mg per kg (body weight). Scales were collected 3 hours later. For label-retaining studies, animals were injected with BrdU twice a day for 1-week, and ‘chased’ (left to metabolize the BrdU in their system) for up to 8 weeks for chickens and 16 weeks for alligators. One chicken and one alligator were euthanized after 1 week’s BrdU labelling (1-week pulse). Four adult chickens and four juvenile alligators were used for our LRC study. For chicken feathers, contour feathers (in both growth and resting phases) on the dorsal region were used. BrdU staining was performed according to^45^.

### Scale wound healing and regeneration

For scale wounding and regeneration, chickens were anesthetized by an intramuscular injection of ketamine (50 mg/kg) and xylazine (5 mg/kg). Alligators were anesthetized by inhaling of 2% isoflurane. Biopsy areas (1 cm wide and 1 cm long, cover at least two scales) were initially drawn with a pen and this line was traced with a scalpel to about 1 mm in depth. The skin was lifted with forceps and excised with a scalpel. Animals were euthanized after 16 weeks regeneration. Paraffin sections were prepared for H&E staining and immunostaining. Two adult chickens and two juvenile alligators were used.

### Double labeling of LRCs and TA with IdU and CldU after alligator skin wounding

IdU (Sigma) was used to label LRCs and CldU (Sigma) was used to label TA cells during skin regeneration. Animals were injected with IdU intraperitoneally (50 mg/kg) two times per day for 1 week followed by a 16-week chase period. A piece of ventral skin (1 cm wide and 1 cm long) was removed. Four days after wounding, animals were injected with IdU (50 mg/kg) for 3 h before being euthanized. Two alligators were used in this study.

For IdU/CldU double staining, CldU was detected using a rat anti-BrdU antibody (BU 1/75; Ab6326-250; Abcam); Biotinylated Goat anti Mouse IgG (Abcam) and Streptavidin (Jackson ImmunoResearch) were used as secondary and tertiary antibodies; AEC (Vectorlabs) was used to develop the red color. IdU was detected by mouse anti-BrdU (347580; BD); Anti-mouse AP (Abcam) was used as secondary antibody; NBT/BCIP (Promega) was used to visualize positive staining (blue). For fluorescent IdU/CldU staining, sections were treated with 0.01 M Citrate buffer (pH 6.0) by microwaving for 6 min. Alexa Fluor anti–mouse-546 (A11030) and anti–rat-488 (A11006) from Invitrogen were used as secondary antibodies. DAPI was used to visualize the nuclei. Stained sections were imaged with an AxioImager (Zeiss).

### Whole mount/section *in situ* hybridization and immunostaining

β-catenin and Shh RNA probes from chicken^46^ and alligator^33^ were used for whole mount *in situ* hybridization. To generate Alligator K15 and K75 RNA probes, PCR was performed using cDNA from Stage 24 alligator skin. Primers for K15, Sense: TGGCCTACCTSAASAAGAAC, antisense: GGTASGTGGCRATCTCCTG. Primers for K75, Sense: GAAGAKGAGATCAACAAGCG; Antisense: GCCAGCTTGACRTTCATCAG. Primers for PCR were designed based on K15 mRNA sequences from chicken (NM_001001312) and green anole (Anolis carolinensis, XM_003222575), and K75 mRNA sequences from chicken (NM_001001314) and green anole (XM_003216971). PCR products were inserted to p-drive (Qiagen) to make antisense RNA probes. Whole mount and section *in situ* hybridization were performed according to procedures described in^47^. Diluted eosin was used for faint counter-staining. Immunostaining (Tenascin-C) was performed according to^33^. β-keratin antibody was from Dr. Roger Sawyer. Alexa Fluor anti–rabbit-488 (A11008) from Invitrogen was used as secondary antibodies.

### Ethical approval and informed consent

All procedures were approved by the local Institutional Animal Care and Use Committee at the University of Southern California, and the Louisiana Department of Wildlife and Fisheries. The methods were carried out in accordance with the relevant guidelines and regulations.

## Electronic supplementary material

Supplementary data
