# Supplementary material for: Comprehensive molecular and cellular studies suggest avian scutate scales are secondarily derived from feathers, and more distant from reptilian scales
Source: Sci Rep. 2018 Nov 13;8:16766. doi: 10.1038/s41598-018-35176-y (PMC6233204; doi:10.1038/s41598-018-35176-y)
Supplement: Supplementary file 1 — Supplementary data [file 41598_2018_35176_MOESM1_ESM.pdf]

Comprehensive molecular and cellular studies suggest avian scutate scales are secondarily derived from feathers, and more distant from reptilian scales

Ping Wu[1], Yung-Chih Lai[1,2], Randall Widelitz[1], Cheng-Ming Chuong[1,2,3*]

[1]Department of Pathology, Keck School of Medicine, Univ. Southern California, Los Angeles, CA 90033

[2]Integrative Stem Cell Center, China Medical University Hospital, China Medical University, Taichung 40402, Taiwan

[3]International laboratory for Wound Repair and Regenerative Research, Graduated Institute of Clinical Medicine, National Cheng Kung University, Tainan, Taiwan.

Supplementary

Figure. S1. List of 86 annotated alligator genes among the 102 chicken *feather-associated genes*

ADAM12, CCDC170, GGT5, KLHL4, NTN3, RUSC2, TEK, ADAMTSL2, GLIS1, LIMCH1, OSR1, SATB2, ADCY5, CLGN, EPB41L3, LOXL3, SIM1, TLN2, AFF3, CPLX2, EPHA5, HBE, LRIG1, PAMR1, LC22A16, TMTC1, CPXM2, ESM1, HPGD, LRRC16A, PDE1A, SMOC1, Ahrr, DNAH10, HRH2, LRRC4C, PGBD5, SOX18, TPD52L1, ALCAM, DRAXIN, FAT3, HSPA4L, Lrrn1, PGM5, ST8SIA6, TSPO2, ANO1, EFEMP1, FBLN1, IGF2, PHLDB2, STEAP3, UBASH3B, ARHGAP25, EMX2, FLNB, JUN, MMEL1, PPP4R4, STK10, ZIC1, ARHGEF3, foxp2, KCNH5, MSANTD1, RAI2, STRA6, ZIC3, FRMD3, KCNS2, MYOCD, RGMA, SYNDIG1, ZP2, BEND5, FST, KIAA2022, NEFM, RND3, SYT6, GAD2, KIFAP3, NT5E, RUNX3, TBX15

Figure. S2. List of 135 annotated alligator genes among the 170 chicken *scale-associated genes*

GSN, FKBP9, ATF4, COL2A1, MDK, COL16A1, SCARF2, PPM1D, HIF1A, MEOX2, SNCAIP, AQP1, P4HA3, FKBP7, SVEP1, FAM101B, PIEZO2, ADAMTS3, SLIT2, WBSCR17, SELM, GTF2IRD1, CACNA1G, HTRA3, FAM46A, BCL6, LMO4, ST3GAL1, CD82, CHST15, IGFBP2, IGSF10, PLBD2, FKBP14, TBX4, TMEM47, NPTX2, NDRG1, RNF34, PRKD1, PANK1, TSHZ1, DVL1, CDH13, ARSI, TLE1, WASF3, IL17REL, KIAA0226L, TPCN1, DACT2, SULT1E1, ATCAY, SLITRK4, NFKBIA, TMEM132E, PDLIM5, KIAA0513, TMEFF2, TPST1, NFATC2, KLHL25, DOCK5, TRIM47, SOHO-1, ZFPM1, DCLK2, SFRP4, COL17A1, FRMPD4, KCNMB4, ASTN1, ELFN1, PITPNM3, PRDM6, CDH1, CDKL1, MDGA2, ATP6AP1L, PLCB1, NTNG1, SHOX, GALNT5, GLRA4, RTN4RL1, PROX1, PKP2, PITX1, LHX9, IL18, PIK3R5, DACH1, CADM2, NT5C1A, NPAS2, SBK1, ADARB2, CHAC1, OPRD1, GABRA1, PI16, RHOV, ASPHD2, TPH2, TFAP2B, CDKN2C, VWC2, STK32A, DRD5, PPM1H, TOX, MOV10L1, RCAN2, PYROXD2, GPR143, Wnt10a, SCN4B, CALCR, MGAT4C, SLC17A9, VGLL1, PRDM12, PTPRO, TMEM215, TEKT5, AJAP1, GLIS3, Wnt3, NRG3, TRPM6, GRIN2C, ADCYAP1R1, SLC34A2, KY, Gnot1
